# Dr. J. V C. Smith of Boston

**Published:** 1851-10

**Authors:** 


### DR. J. V. C. SMITH, OF BOSTON.

We cordially welcome the return of this confrère, the veteran editor of the Boston Medical and Surgical Journal, who has just completed an extensive tour through Europe, Asia, and Africa, and is entering again upon his arduous labors. After much intercourse with medical men in the old world he thus speaks of his professional brethren at home:—

"Diseases are nowhere controlled with more skill than in the United States, nor is there a country on the globe where the benefits accruing to society from the efforts of indefatigable practitioners of medicine and surgery, are more apparent. If further advances have been made in Europe, they have resulted from a sub-division of labor. Those most distinguished for their accuracy in detecting the causes of disease, and in the administration of remedies, have attained their tact and distinction by pursuing a specialty"

